# Supplementary material for: Conditioned Medium of Intervertebral Disc Cells Inhibits Osteo-Genesis on Autologous Bone-Marrow-Derived Mesenchymal Stromal Cells and Osteoblasts
Source: Biomedicines. 2024 Feb 6;12(2):376. doi: 10.3390/biomedicines12020376 (PMC10886592; doi:10.3390/biomedicines12020376)

Figure S1. Col2/Col1 gene expression ratio of donor-matched primary cell types at passage 2-4 in OB, MSC, AFC, CEPC, and NPC. The graph clearly demonstrates that AFC, CEPC and NPC in increasing order do express higher levels of col2, which is absent in OBs and MSCs. Mean ± SD, N = 3 donors.

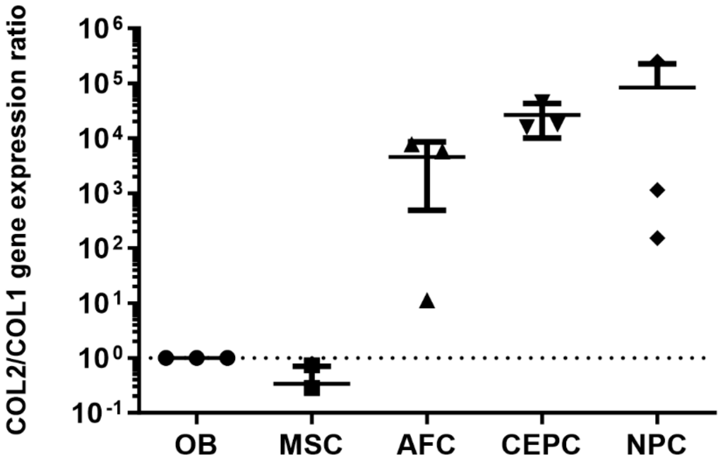

Supplement: Supplementary file 1 [file biomedicines-12-00376-s001.zip › biomedicines-2797419-supplementary.pdf]
